# A PDMS–Agar Hybrid Microfluidic Device for the Investigation of Chemical–Mechanical Associative Learning Behavior of *C. elegans*

**DOI:** 10.3390/mi14081576

**Published:** 2023-08-10

**Authors:** Jinchi Zhu, Yu Wang, Shuting Tang, Huiying Su, Xixian Wang, Wei Du, Yun Wang, Bi-Feng Liu

**Affiliations:** 1 School of Bioengineering, Huainan Normal University, Huainan 232038, China; 2 The Key Laboratory for Biomedical Photonics of MOE at Wuhan National Laboratory for Optoelectronics-Hubei Bioinformatics & Molecular Imaging Key Laboratory, Systems Biology Theme, Department of Biomedical Engineering, College of Life Science and Technology, Huazhong University of Science and Technology, Wuhan 430074, China; 3 Department of Cell Biology, College of Medicine, Jiaxing University, 118 Jiahang Road, Jiaxing 314001, China

**Keywords:** associative learning behavior, microfluidic device, *Caenorhabditis elegans*, octopamine

## Abstract

Associative learning is a critical survival trait that promotes behavioral plasticity in response to changing environments. Chemosensation and mechanosensation are important sensory modalities that enable animals to gather information about their internal state and external environment. However, there is a limited amount of research on these two modalities. In this paper, a novel PDMS–agar hybrid microfluidic device is proposed for training and analyzing chemical–mechanical associative learning behavior in the nematode *Caenorhabditis elegans*. The microfluidic device consisted of a bottom agar gel layer and an upper PDMS layer. A chemical concentration gradient was generated on the agar gel layer, and the PDMS layer served to mimic mechanical stimuli. Based on this platform, *C. elegans* can perform chemical–mechanical associative learning behavior after training. Our findings indicated that the aversive component of training is the primary driver of the observed associative learning behavior. In addition, the results indicated that the neurotransmitter octopamine is involved in regulating this associative learning behavior via the SER-6 receptor. Thus, the microfluidic device provides a highly efficient platform for studying the associative learning behavior of *C. elegans*, and it may be applied in mutant screening and drug testing.

## 1. Introduction

Learning and memory are significant characteristics that enable animals to adapt to their changing environments [1]. Associative learning, a form of behavior plasticity [2], involves associating one behavior or stimulus (conditioned stimulus, CS) with another stimulus (unconditioned stimulus, US) [3]. Various stimuli, including olfactory, gustatory, auditory, visual, and temperature-sensing stimuli, have been identified in associative learning [4,5,6,7,8]. Associative learning, which was first demonstrated by Pavlov (1927), has been reported in a wide range of animals, from snails and *Drosophila* to nematodes, honeybees, and rats [9,10,11,12,13]. Through associative learning, animals can seek advantages and avoid harm, thereby enhancing their chance of survival.

*Caenorhabditis elegans* (*C. elegans*) is a hermaphroditic, free-living nematode that feeds on bacteria, lives in soil, and poses no harm to humans. *C. elegans* is an ideal biological model system for use in various fields of life sciences, including cell biology, neuroscience, developmental biology, and aging [14,15]. Due to its relatively simple nervous system (302 neurons), *C. elegans* has frequently been chosen for studying individuals’ learning and memory [16,17]. For example, Choi et al. demonstrated that *C. elegans’* learning experience weakened the gap junction and induced an offensive response to a training odor, leading to the uncoupling of the interneurons’ response to the training odor and resulting in learned olfactory behavior [18]. Recently, Chien-Po et al. identified a neuronal circuit that mediated aversive memory under systemic mitochondrial stress in nematodes, and found that octopamine regulated the learned avoidance behavior [19]. Importantly, although the nervous system of a nematode is compact, it is highly homologous with the mammalian system [20]. Therefore, it is highly meaningful to use *C. elegans* as experimental subjects to study learning and memory.

Recently, microfluidic chips [21,22,23], a powerful technology, have been used to conduct scientific research on nematodes [24,25,26]. The main advantages of microfluidic chips for *C. elegans* research include their size compatibility, gas-permeable and transparent materials, high-throughput manipulation with a single-nematode resolution, and convenient, real-time observation under a microscope. Several microfluidic chips have been reported for investigating associative learning behavior in *C. elegans*. For example, Bargmann et al. fabricated a four-choice maze micro-device to investigate olfactory learning in *C. elegans* and found that aversive learning required 5-HT secreted from the ADF neuron and the 5-HT receptor MOD-1 [27]. In addition, a microfluidic T-maze was presented to analyze the associative learning of nematodes, linking a food reward to the maze-choice behavior [28]. Furthermore, our group developed a microfluidic device to investigate the food–mechanosensation associative learning of *C. elegans* [29]. In this microfluidic device, the alternating arrangement of PDMS columns and blank regions were used to simulate mechanical stimuli for the worms. Although food has often been used as an unconditioned stimulus (US) in previous associative learning studies, its effect on *C. elegans* integrates gustatory, olfactory, and mechanical stimuli. In order to enhance the investigation of *C. elegans*’ associative learning behavior and facilitate more precise assessments of this behavior, it may be advantageous to utilize a training approach that involves two unambiguous conditions. 

Animals employ various sensory mechanisms to obtain information about their external environments and internal states, among which chemosensory cues are generally associated with food or danger, and mechanosensation is often used to perceive the surrounding environment, especially for organisms that live in soil and water [30,31,32]. Despite their significance, a few studies have specifically investigated the associative learning behavior between chemosensation and mechanosensation. Therefore, the present study aims to address this gap by utilizing a microfluidic chip to integrate both types of stimuli in a more controlled and precise manner. This method aims to elicit the desired associative learning behavior with greater accuracy and reliability, thereby allowing more meaningful conclusions to be drawn from the experiments.

In this study, we proposed a novel microfluidic device to explore the associative learning behavior between chemosensation and mechanosensation in *C. elegans*. The PDMS–agar hybrid chip was designed to generate the chemical concentration gradient on the agar gel layer and mimic mechanical stimuli on the PDMS layer. By training *C. elegans* using the microfluidic device, we successfully induced chemical–mechanical associative learning behavior in the worms. We further investigated the effect of related neurotransmitters and receptors on the learning behavior, providing new insights into the underlying mechanisms. This microfluidic device provided a unique method for studying associative learning behavior in nematode populations, and it can be become a user-friendly, reliable platform for drug testing and mutant screening.

## 2. Materials and Methods

### 2.1. Materials

MgSO_4_, KH_2_PO_4_, Na_2_HPO_4_·12H_2_O, CaCl_2_, K_2_HPO_4_, NaCl, and ethanol were purchased from Aladdin Biochemical Technology Co., Ltd. (Shanghai, China). Agar, yeast extract, cholesterol, tryptone, biotin, glycerol, and copper acetate were purchased from Sigma-Aldrich (San Diego, CA, USA). SU-8^TM^ (GM 1075) was purchased from Gersteltec Sarl. PDMS Sylgard 184 was purchased from Dow Corning (Midland, MI, USA). Water was purified using a Medium EDI System (HHitech, Shanghai, China). 

### 2.2. Nematode Strains and Culture

A single Escherichia coli (*E. coli*) OP50 colony was aseptically inoculated in 20 mL LB broth, and the inoculated cultures were shaken for 12 h at 37 °C. The *E. coli* OP50 liquid culture was stored at 4 °C. *C. elegans* were cultured at 20 °C on Nematode Growth Medium (NGM) Petri plates that had been seeded with *E. coli* OP50 as food [33]. The NGM preparation steps were shown below. Mix 1.5 g NaCl, 1.25 g peptone, 8.5 g agar, and 487 mL H_2_O in a 1 L flask. Autoclave for 30 min, then cool to 55 °C. Add 0.5 mL 1 M CaCl_2_, 0.5 mL 1 M MgSO_4_, and 0.5 mL 5 mg/mL cholesterol in ethanol and 12.5 mL 1 M KPO_4_ buffer, and mix well. N2 Bristol, RB1161 tbh-1 (ok1196)X, CB1515 mec-10 (e1515)X, DA572 eat-4 (ad572)III, DA1774 ser-3 (ad1774)I, CB1033 che-2 (e1033)X, TU253 mec-4 (u253)X, VC818 trp-4 (gk341)I, CX10 osm-9 (ky10)IV, PR674 che-1 (p674)I, and MT15434 tph-1 (mg280)II strains were kindly provided by the Caenorhabditis Genetics Center. N2 was used as the wild-type (WT) reference strain. The worms were synchronized using a hypochlorite (1% NaClO and 0.5 M NaOH) treatment of gravid hermaphrodites. The obtained eggs were transferred to NGM plate and cultured for about 60 h at 20 °C, until they reached the young adult stage. The behavior of worms was observed using a stereomicroscope.

### 2.3. Chip Design and Fabrication

We fabricated microfluidic chips via standard soft lithography using single-layer SU8 photoresist molds [34]. We deposited a 90 µm-thick layer of SU8 on the silicon wafer. The PDMS mixture was poured onto the SU8 mold, degassed, and cured at 75 °C for 90 min. After curing, a piece of PDMS (~4 mm thick) was cut and separated. A hole (2 mm diameter) was punched on the middle of the PDMS, the entrance of the worms. The device consisted of a bottom agar gel layer and an upper PDMS layer. The composition of the agar layer was NGM. Dispense NGM solution into the special Petri plate. After the medium cooled, seed *E. coli* OP50. The agar layer mainly served two purposes, forming a chemical gradient and providing a suitable substrate for nematodes to crawl freely. After being exposed to oxygen plasma, the surface of PDMS became hydrophilic. Relying on hydrophilicity, PDMS could reversibly bond with agar gel substrate. PDMS–agar hybrid microfluidic devices were developed for training *C. elegans* and analyzing chemical–mechanical associative learning behavior.

**Figure 1:**
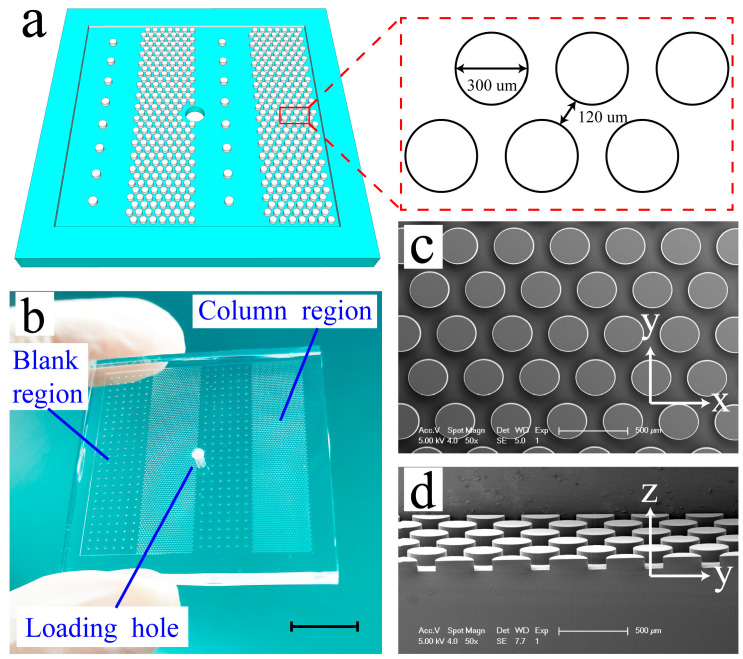
Pictures of the microfluidic device: (**a**) The schematic diagram of the top PDMS layer of the microfluidic device. Inset shows an enlarged view. The column diameter was 300 μm, and the gap between columns was 120 μm. (**b**) An image of the PDMS layer. The scale bar represents 10 mm. (**c**,**d**) A series of SEM images of the column array in the microfluidic device. The scale bars represent 500 μm.

**Figure 2:**
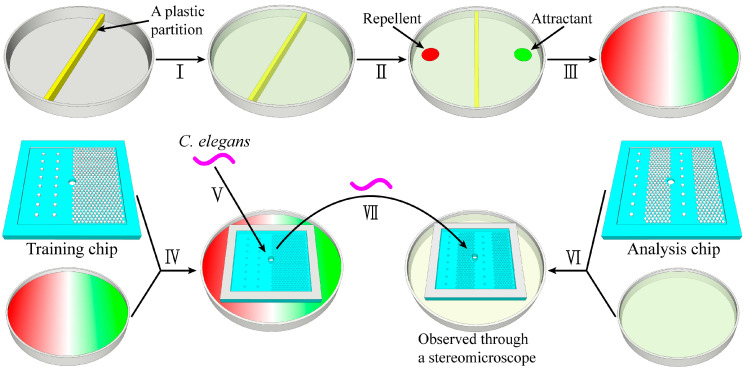
Schematic diagrams and operation procedures: Ⅰ: This Petri plate was specially made with a plastic partition in the middle. Dispense NGM solution into the Petri plate. Seed *E. coli* OP50. Ⅱ: The attractant and the repellent were dripped onto the NGM plate. Ⅲ: They were equilibrated for 24 h to form chemical gradients. Ⅳ: We bonded the training chip onto the NGM plate (with chemical gradients). Ⅴ: Worms were picked and placed into the training chip. Ⅵ: We bonded the analysis chip onto a conventional NGM plate. Ⅶ: After training, *C. elegans* were transferred to the analysis chip. Thirty minutes later, the results were observed using a stereomicroscope.

Our system includes two chips (one for training and one for analysis). The analysis chip is composed of two blank regions and two column array regions (Figure 1b) used for mimicking mechanical stimuli via collision with the columns, which is consistent with our previous study [29]. The column is 300 μm in diameter, 90 μm in height, and the gap between the columns is uniform (120 μm) (Figure 1a,c,d). The blank region contains a little pillar to prevent the chip from collapsing. The training chip, whose dimension is consistent with the analysis chip, consists of one column array region and one blank region (Figure 2: training chip). The training chip was used to train the worms, and the analysis chip was used to analyze the associative learning behavior. These two chips can be cleaned and recycled.

### 2.4. Chemical–Mechanical Associative Learning Behavior Assay

The procedures of assay are shown in Figure 2. Ⅰ: This Petri plate (90 mm diameter) was specially made with a plastic partition in the middle. We dispensed an appropriate amount of NGM solution into the Petri plate, so that the liquid level slightly overflowed the middle partition. After the medium cooled, we seeded *E. coli* OP50 onto the NGM plate, and allowed it to grow overnight at room temperature. Ⅱ: Then, 30 μL of attractant (0.2 M biotin solution) was dripped onto the NGM plate, 15 mm away from the partition, and 30 μL of repellent (0.1 M copper acetate solution) was dripped on the other side of the partition. Ⅲ: The plate was allowed to equilibrate for 24 h, forming chemical gradients. Ⅳ: We bonded the training chip onto the NGM plate (with chemical gradients), with the column region on the side of the attractant. Ⅴ: About 40 synchronized worms were put into the PDMS–agar hybrid microfluidic device through the loading hole with a worm picker. We transferred the training micro-device to the incubator (20 °C), and the worms were trained on the chip for 24/48 h. Ⅵ: We bonded the analysis chip onto a conventional NGM plate. Ⅶ: After training, the worms were transferred to the analysis chip to investigate the learning behavior. Thirty minutes later, the results were recorded using a stereomicroscope. Each experiment had to be performed three times.

The behavior of *C. elegans* was quantified via a binary choice assay, in which the worms crawled towards one of the two regions on the analysis chip. The choice and learning indices were based on Bargmann’s research [27]. Choice Index = (NWC − NWB)/TN. The NWC indicated the number of *C. elegans* in the column region, the NWB indicated the number of *C. elegans* in the blank region, and the TN indicated total number of *C. elegans* on the analysis chip. In this experiment, a choice index of 1.0 represented a complete preference for the column array region, an index of −1.0 represented a complete preference for the blank region, and an index of 0 represented an equal distribution. Learning Index = Choice Index of trained worms − Choice Index of naive worms. A positive learning index represented that the trained worms exhibited associative learning behaviors.

## 3. Results and Discussion

### 3.1. Construction of Chemical Gradient

**Figure 3:**
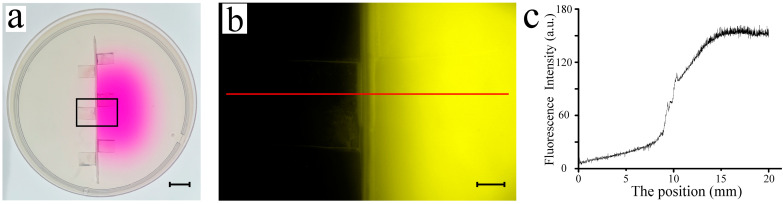
Evaluation of the chemical gradient: (**a**) First, 30 μL of Rhodamine B solution was dripped on the NGM plate at 15 mm on the right side of the middle partition. Twenty-four hours later, we obtain a panoramic picture of the plate. Scale bar: 10 mm. (**b**) Fluorescence image of the NGM, corresponding to the black wireframe in (**a**). Scale bar: 2 mm. (**c**) The fluorescence intensity profile, corresponding to the red line in Figure (**b**).

A fluorescent solution (Rhodamine B, 0.1 mg/mL) was used to validate the chemical gradient on the NGM plate, and the results are shown in Figure 3. Our custom Petri dish (90 mm diameter) featured a plastic partition in the center (Figure 3a). Rhodamine B solution (30 μL) was dripped on the NGM plate at 15 mm to the right of the middle partition. The plate was equilibrated for 24 h at 4 °C to prevent excessive drying. As shown in Figure 3a, Rhodamine B diffused through the agar gel; however, this diffusion was halted by the middle partition. Fluorescence intensity was captured using an OLYMPUS SZX16 fluorescent microscope (Figure 3b). The fluorescence profile of the NGM was plotted as a function of position (Figure 3c). It can be seen that the right-side fluorescence intensity was considerably higher than that on the left, indicating that the partition design was effective in preventing diffusion. Overall, the chemical gradient could be formed by using this specialized Petri dish.

### 3.2. Associative Learning Behavior in Chip

Our previous investigation demonstrated that the micro-column array could emulate mechanical stimuli, allowing the worms to perform associative learning behavior (between food and mechanosensation) through training on the chip. In this study, we further developed a novel microfluidic device to investigate chemosensation and mechanosensation in *C. elegans*. A total of 0.2 M biotin solution and 0.1 M copper acetate solution were selected as the attractant and repellent, respectively. *C. elegans* N2 (wild type) were used as experimental objects. The experimental group was the worms trained on the training chip, while the control group included the worms maintained on the conventional NGM plate. The experimental procedure involved the steps outlined above.

**Figure 4:**
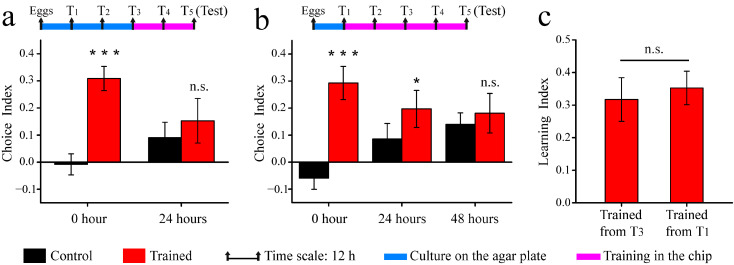
The associative learning ability and memory ability of wild-type *C. elegans*: (**a**) Choice index of worms trained from T3 to T5, tested at different times (0 h and 24 h after training). (**b**) Choice index of worms trained from T1 to T5, tested at different times (0 h, 24 h, and 48 h after training). (**c**) Learning index of worms trained for different periods, tested at 0 h after being trained. Data are displayed as means ± SEM (n = 3 assays). n.s., not significant. * *p* < 0.05, *** *p* < 0.001.

The learning behavior of *C. elegans* was assessed for various training durations (from T1 to T5; and from T3 to T5), with 12 h intervals between each stage (Figure 4a,b). The developmental stage T5 corresponded to young adult hermaphrodites. In the training chip, the column region was bonded to the attractant side. After training, the worms were tested on the analysis chip. As shown in Figure 4a,b, the trained worms exhibited a stronger inclination toward the column region compared with that of the untrained worms. These finding indicated that *C. elegans* successfully performed chemical–mechanical associative learning behavior after being trained on the microfluidic device, validating that our micro-device was functional.

Figure 4c demonstrates the result of the learning index for different training durations. However, minimal differences were observed between the two conditions. The results suggested that the associative learning ability did not improve with an extended training time, which is likely because the worms (T1–T3) were too small to sufficiently collide with the column. In Figure 4a,b, the memory ability of the trained worms was also evaluated at different times (24 h after training; and 48 h after training). We found that the memory ability was virtually non-existent for this complex chemical–mechanical associative learning behavior.

### 3.3. Associative Learning Behavior of Mechanosensory and Chemosensory Mutants

**Figure 5:**
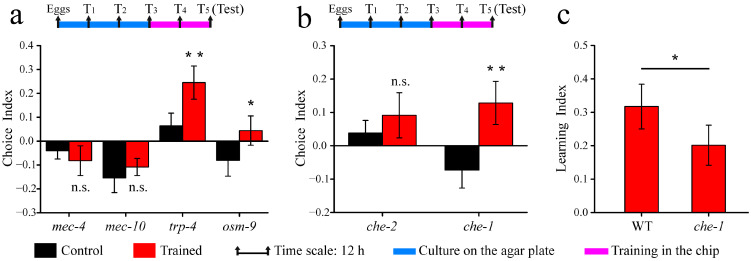
Associative learning behavior of mechanosensory and chemosensory mutations: (**a**) The choice index of mutations in mechanosensitivity. (**b**) The choice index of chemosensory mutations. (**c**) The learning index of wild-type and *che-1* mutation. Data are displayed as means ± SEM (n = 3 assays). n.s., not significant. * *p* < 0.05; ** *p* < 0.01.

Three sensory systems, namely, proprioception, nose touches, and body touches, are included in the mechanosensation of *C. elegans* [35]. To explore the mechanism of mechanical stimuli in associative learning behavior, four mutants (*trp-4*, *mec-4*, *mec-10,* and *osm-9*) were tested. As shown in Figure 5a, the spatial distribution in the analysis of *mec-4* and *mec-10* mutants showed negligible differences between the trained and untrained groups. The finding suggested that *mec-10* and *mec-4* mutants were deficient in this associative learning behavior. Upon training, *trp-4* and *osm-9* mutants exhibited some learning abilities; however, their learning capabilities were not remarkable (Figure 5a). According to previous research, MEC-4 and MEC-10 channel proteins are necessary for gentle touch mechanosensation [36,37], *osm-9* mutations result in nose touch insensitivity [38], and the TRP-4 protein is associated with stretch-receptor-mediated proprioception [39]. The above results suggested that gentle touch is crucial for the observed chemical–mechanical associative learning behavior, while proprioception and nose touch only have a fractional effect on their learning ability.

To investigate the role of chemosensation in this associative learning behavior, *che-2* and *che-1(p674)* mutants were tested. The *che-2* mutant, which exhibits abnormal chemotaxis, displayed no obvious preference for the column region following training (Figure 5b), indicating that chemosensation is crucial for this associative learning behavior. In contrast, the *che-1(p674)* mutant displayed the learning behavior (Figure 5b). The learning index of the *che-1(p674)* mutant was slightly lower than that of the wild-type one (Figure 5c). The *che-1(p674)* mutant was defective in chemotaxis to all attractants except pyridine and D-tryptophan, but its avoidance response to chemical repellents, such as copper, was normal [40]. The results indicated that the aversive component of learning constitutes the core element of this associative learning behavior.

### 3.4. Associative Learning Behavior of Neurotransmitter and Receptor Mutants

**Figure 6:**
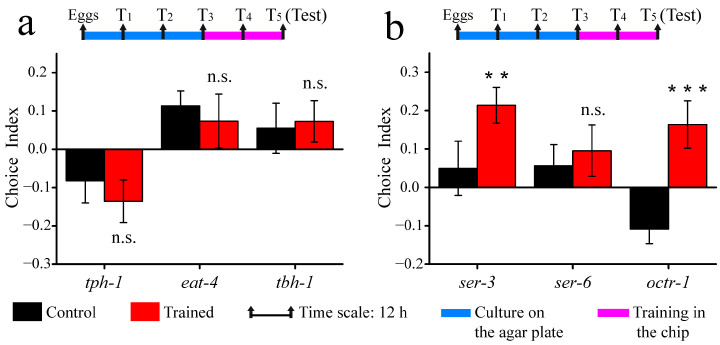
Associative learning behavior of neurotransmitter and receptor mutations: (**a**) The choice index of mutations in neurotransmitter. (**b**) The choice index of mutations in receptor. Data are displayed as means ± SEM (n = 3 assays). n.s., not significant. ** *p* < 0.01; *** *p* < 0.001.

A variety of neurotransmitters modulate behaviors in response to changing environmental cues for *C. elegans*. To investigate the role of neurotransmitters in this associative learning behavior, *tph-1*, *eat-4,* and *tbh-1* mutants were tested. As shown in Figure 6a, all three mutations in serotonin synthesis (*tph-1*), glutamate transporter (*eat-4*), and octopamine synthesis (*tbh-1*) were found to result in defective learning, suggesting that the above three neurotransmitters are essential for associative learning.

Previous studies had shown that octopamine promotes aversive olfactory learning in *Drosophila* [41]. To further investigate octopamine modulation, we examined *C. elegans* mutations of GPCRs for octopamine, and the results are presented in Figure 6b. Compared with wild-type *C. elegans*, *octr-1* and *ser-3* mutants displayed a preference for the column region, while *ser-6* mutants did not exhibit learning abilities. Taken together, these findings suggested that octopamine exerts its effects in the chemical–mechanical associative learning through the SER-6 receptor.

## 4. Conclusions

In this study, we developed a PDMS–agar hybrid microfluidic device that established chemical–mechanical associative learning behavior in *C. elegans*. The agar gel layer allowed the generation of a chemical concentration gradient, while the PDMS layer, composed of blank and column array regions, was used to produce mechanical stimuli. By using this device, we observed that wild-type *C. elegans* were able to effectively establish associative learning behaviors, whereas they had poor memory retention. Further, the mechanosensory mutant tests indicated that gentle touch mechanosensation plays a crucial role in chemical–mechanical associative learning. In terms of chemosensation, the results revealed that the aversive component of learning is the core element. Finally, our exploration of the role of neurotransmitters and receptors indicated that multiple neurotransmitters regulate this associative learning behavior, and octopamine acts through the SER-6 receptor. Our platform is inexpensive, recyclable, and convenient to operate. It also has the potential to become a user-friendly, reliable tool for drug testing and mutant screening. In conclusion, our study provides new insights into the mechanisms of associative learning in *C. elegans* and highlights the potential of our micro-device as a valuable tool for future research in this area.

## Data Availability

The data presented in this study are available upon request from the corresponding author.
